# TREM-1 Modulation Strategies for Sepsis

**DOI:** 10.3389/fimmu.2022.907387

**Published:** 2022-06-15

**Authors:** Sara Siskind, Max Brenner, Ping Wang

**Affiliations:** ^1^ Center for Immunology and Inflammation, The Feinstein Institutes for Medical Research, Manhasset, NY, United States; ^2^ Department of Surgery, Zucker School of Medicine at Hofstra/Northwell, Manhasset, NY, United States; ^3^ Elmezzi Graduate School of Molecular Medicine, Manhasset, NY, United States

**Keywords:** sepsis, inflammation, TREM-1, shock, DAMP

## Abstract

The triggering receptor expressed on myeloid cells-1 (TREM-1) is a pattern recognition receptor, which can be upregulated in inflammatory diseases as an amplifier of immune responses. Once activated, TREM-1 induces the production and release of pro-inflammatory cytokines and chemokines, in addition to increasing its own expression and circulating levels of the cleaved soluble extracellular portion of TREM-1 (sTREM-1). This amplification of the inflammatory response by TREM-1 has now been considered as a critical contributor to the dysregulated immune responses in sepsis. Studies have shown that in septic patients there is an elevated expression of TREM-1 on immune cells and increased circulating levels of sTREM-1, associated with increased mortality. As a result, a considerable effort has been made towards identifying endogenous ligands of TREM-1 and developing TREM-1 inhibitory peptides to attenuate the exacerbated inflammatory response in sepsis. TREM-1 modulation has proven a promising strategy for the development of therapeutic agents to treat sepsis. Therefore, this review encompasses the ligands investigated as activators of TREM-1 thus far and highlights the development and efficacy of novel inhibitors for the treatment of sepsis and septic shock.

## Introduction

Sepsis is a complex disorder, defined by a dysregulated host response to infection leading to organ dysfunction (1, 2). The pro-inflammatory response to invading pathogens is initiated by pattern recognitions receptors (PRRs) located on the surface and intracellularly of immune and non-immune cells, that recognize pathogen-associated molecular patterns (PAMPs), molecules released from pathogens, and damage-associated molecular patterns (DAMPs), molecules released from damaged cells (3, 4). Activation of PRRs on innate immune cells initiates microbicidal and pro-inflammatory responses to contain and eliminate the invading pathogens and activates the adaptive immune response, particularly T lymphocytes (3). Toll-like receptors (TLRs) are a well-studied family of PRRs for their role in innate immunity that amplify the inflammatory response synergistically with triggering receptor expressed on myeloid cells-1 (TREM-1), a more recently characterized PRR (5–8).

TREM-1 was first identified on circulating neutrophils and monocytes in response to lipopolysaccharide (LPS), a glycan mostly present on the surface of Gram-negative bacteria that strongly activates the host immune response (9–11). TREM-1 is part of a family of TREM receptors that also includes TREM-2, TREM-3, and TREM-like transcript-1 and 2 (TLT-1, TLT-2) (12). TREM-3 is only expressed in mice, and like TREM-1, is upregulated in response to LPS. TREM-2, on the other hand, is downregulated in response to LPS and, upon activation, attenuates the inflammatory response (12, 13). Activation of TREM-1 in neutrophils and monocytes triggers the release of pro-inflammatory cytokines and chemokines, in addition to upregulating the gene expression of TREM-1 and surface expression of cell activation markers (9). This amplification of the inflammatory response by TREM-1 has gained interest as a critical contributor to the dysregulated immune response in sepsis (4). Patients admitted to the ICU with septic shock have higher surface expression of TREM-1 on monocytes and elevated circulating levels of the cleaved soluble extracellular portion of TREM-1 (sTREM-1) (14, 15). Elevated levels of circulating sTREM-1 have also been associated with increased mortality in patients with septic shock (16, 17). Additionally, mice genetically deficient in TREM-1 had less severe disease in response to multiple pathogens without affecting their ability to clear the infection, resulting in similar pathogen loads (18). The inhibition of TREM-1 remains a promising target for development of therapeutic agents to treat sepsis. This review aims to give an overview of the role of TREM-1 and its endogenous ligands in sepsis, and the development of novel inhibitors of this pathway and their efficacy for the treatment of sepsis.

## Structure and Signaling Pathway

TREM-1 is a member of the immunoglobin superfamily, a group of cell surface receptors with related extracellular Ig-like domains (19, 20). In addition to the extracellular Ig domain, it consists of a transmembrane region with a conserved lysine residue, and a short cytoplasmic domain that does not contain a signaling motif (21, 22). Propagation of signaling is instead dependent on association with the immunoreceptor adaptor protein DNAX activation protein 12 (DAP12). TREM-1 contains a positively charged transmembrane lysine residue that associates with a negatively charged aspartate residue of DAP12 (21–24). Upon receptor activation, the immunoreceptor tyrosine-based activation motif (ITAM) of DAP12 is phosphorylated, signaling the recruitment and activation of spleen tyrosine kinase (Syk), a nonreceptor tyrosine kinase (20). Syk activates multiple downstream signal transduction pathways including the PI3K/Akt pathway, the Ras/ERK/MAPK pathway, NF-κB signaling, and phospholipase C phosphorylation, leading to increased intracellular calcium and proinflammatory cytokine secretion (19–22, 25).

TREM-1 activation independently triggers downstream inflammatory cascades that can synergize with TLR signaling pathways (22, 25). TLR4 activation upregulates the expression of TREM-1, and the concomitant activation of TREM-1 and TLR4 leads to a synergistic increase in proinflammatory cytokine and chemokine release by 25-fold compared with that of TLR4 activation alone (8, 26–28). One proposed mechanism for their synergistic proinflammatory response is that TREM-1 increases the availability of TLR4 downstream signaling molecules such as MyD88, CD14, NF-κB, and IκBα (26). When TREM-1 s blocked, RAW cells stimulated with LPS maintain TLR4 expression but have decreased genetic expression of these signaling molecules as well as inflammatory cytokines (21, 26, 29). Understanding the structure and signaling pathway of TREM-1 remains an important focus of research to better elucidate its role in sepsis and to develop novel therapeutic targets.

## TREM-1 Ligands

The identification of TREM-1’s endogenous ligands is crucial for studying the role of TREM-1 in the pathogenesis of sepsis. Moreover, knowing TREM-1’s endogenous ligands and their structure offers critical insights to develop TREM-1-targeting pharmacological strategies to reduce sepsis hyperinflammation, such as the inhibitory peptides discussed below. Although TREM-1 was characterized in 2000, it took 14 years until the first ligand was identified. Since then, multiple ligands have been implicated as activators of TREM-1, furthering our knowledge on this receptor’s role in innate immunity, sepsis, and non-infectious inflammatory diseases.

### Peptidoglycan Receptor Protein 1 (PGLYRP1)

PGLYRP1 (also named Tag7) was one of the first identified ligands of TREM-1 (30). PGLYRP1 is an antimicrobial protein that is secreted from polymorphonuclear leukocyte granules in response to infection (5, 30, 31). It subsequently binds to peptidoglycan and LPS, essential components of the bacterial membrane, where it induces lethal membrane depolarization and oxidative stress to the bacteria (32–34). After it was discovered that an endogenous, unknown ligand of TREM-1 exists on bacterially activated peritoneal neutrophils, PGLYRP1 was investigated as a potential ligand (30, 35). Neutrophils were stimulated with peptidoglycan and cross-linked to sTREM-1 and, using mass spectrometry, identified that the resulting complexes contained PGLYRP1 peptides (30).

PGLYRP1 as a ligand for TREM-1 was further studied using affinity chromatography, which demonstrated conclusively that immobilized soluble TREM-1 binds PGLYRP1 (36). It has also been shown that PGLYRP1 is an activating ligand of TREM-1 on monocytes and induces the development of cytotoxic lymphocyte subpopulations in peripheral blood mononuclear cells (PBMCs) (37). In the presence of LP17, an inhibitory peptide of TREM-1 discussed further below, monocytes treated with PGLYRP1 failed to induce cytotoxic lymphocyte transformation and had reduced secretion of IL-2, thus providing further evidence that PGLYRP1 is an endogenous ligand for TREM-1 (36, 37). Interestingly, soluble PGLYRP1 alone does not activate TREM-1. Only when complexed with peptidoglycan, anchored to HEK293 cell surface, or bound to a plate, was PGLYRP1 able to elicit an inflammatory response *via* TREM-1 (30). This should be kept in mind for future research on PGLYRP1/TREM-1 interaction, as activation may only be reproducible when TREM-1 is bound to a cell surface.

### Extracellular Cold Inducible RNA Binding Protein (eCIRP)

eCIRP is a DAMP that has recently been identified as an endogenous ligand of TREM-1. It was originally discovered in the blood of critically ill septic and trauma-hemorrhage surgical patients. CIRP is a ubiquitously expressed nuclear protein which has been mostly studied in macrophages, lymphocytes, and neutrophils, which release it into circulation during periods of hypoxic and mild hypothermic stress, such as in sepsis (38, 39). Mice injected with recombinant murine CIRP (rmCIRP) developed a sepsis-like acute lung injury with vascular endothelial cell damage, leukocyte infiltration and increased pro-inflammatory cytokine production (40). Using surface plasmon resonance (SPR), it was determined that rmCIRP binds to recombinant murine TREM-1 (rmTREM-1) with strong affinity, with a *K_D_* of 11.7 × 10^-8^ M (41). Binding was further demonstrated between rmCIRP and surface TREM-1 on murine RAW264.7 and peritoneal macrophages using fluorescence resonance energy transfer (FRET) analysis. rmCIRP-injected mice had attenuated systemic and pulmonary inflammatory response after treatment with the TREM-1 inhibitor LP17. Additionally, mice deficient in TREM-1 had reduced serum IL-6 and IL1-β after rmCIRP injection, indicating eCIRP acts an endogenous ligand of TREM-1 (41).

### High Mobility Group Box 1 (HMGB1)

HMGB1 has also been investigated as an endogenous ligand to TREM-1 (42). HMGB1 was originally identified as a nuclear DNA-binding protein that functions as a cofactor in transcription regulation. It was later found that it can be released from a variety of cells and function as a DAMP, signaling through activation of TLRs and receptor for advanced glycation end products (RAGE) to induce a pro-inflammatory response (43–45). More recently, using a murine model of hepatocellular carcinoma, HMGB1 released from necrotic hepatocytes was found to bind TREM-1 using immunoblotting and SPR, with a binding K_D_ of 35.4 x 10^-6^ M (42, 46). HMGB1 has also been shown to induce inflammatory responses through TREM-1 activation in THP-1 human monocytic cells, a human monocyte cell line, and upregulate TREM-1 expression on macrophages (47, 48). However, recombinant HMGB1 alone was insufficient to induce TREM-1-regulated M1 polarization, suggesting it may need co-activating molecules to fully trigger TREM-1, or that recombinant HMGB1 is different from endogenous HMGB1 (33, 49).

### Heat Shock Protein 70 kDa (Hsp70)

Along with HMGB1, the 70 kDa heat shock protein (Hsp70) was found to be released from necrotic cells and augment the proinflammatory response through TREM-1 activation (42, 47). Under normal conditions, HSP70 is a molecular chaperone that participates in the maintenance of protein homeostasis through folding and remodeling processes (50). Normally expressed at low levels, Hsp70 becomes upregulated in pro-inflammatory states like bacterial endotoxemia (47). In addition to Hsp70’s primary role of protein folding stabilization, there has been growing interest in its function as a pro-inflammatory mediator after it was found to upregulate TNF-α, IL-1β, and IL-6 in human monocytes (51). Hsp70 was then investigated as a novel ligand for TREM-1 using LPS- and necrotic cell lysate-stimulated THP-1 cells. These cells, when treated with an anti-HSP70 antibody, had a reduction in expression of TNF-α, IL-6, and IL-8 that was further reduced by the addition of an inhibitory recombinant TREM-1 fusion chimera (47). This data suggests that Hsp70 is released from necrotic cells to aid pro-inflammatory responses in monocytes through TREM-1 activation of the cytokine expression cascade (47). Hsp70 as a ligand of TREM-1 was further studied using affinity chromatography and demonstrated to bind to sTREM-1 immobilized on a CNBr-Sepharose column as well as to TREM-1 on the monocyte surface (52). Interestingly, however, it was found that Hsp70 released from necrotic Kupffer cells along with HMGB1 in a murine model of hepatocellular carcinoma, does not directly bind to TREM-1, suggesting that HMGB1 may have a stronger affinity and compete for binding on TREM-1 (42).

### Extracellular Actin

Actin is one of the most abundant proteins within cells, functioning in the form of filaments that polymerize to aid in cell morphology and motility (53). When cells undergo apoptosis and necrosis, such as during sepsis, actin is released and has deleterious effects once extracellular (53). After it was determined that an unknown ligand expressed on platelets activates TREM-1 in sepsis, further investigation using gel analysis of platelet total protein and rTREM-1 suggested the ligand to be actin (54, 55). Confocal microscopy was used to confirm the co-localization of TREM-1 and actin on RAW267.7 cells treated with LPS-stimulated platelets and LPS with recombinant actin. This co-localization was also demonstrated *in vivo* using a CLP model of polymicrobial sepsis. Actin and TREM-1 had increased expression and co-localized in the lungs of septic mice (55). Additionally, actin dose-dependently enhanced LPS-stimulated release of TNF-α from RAW267.7 cells and peritoneal macrophages (55). This response was blunted in cells treated with the TREM-1 inhibitor LP17 as well as in peritoneal macrophages isolated from TREM-1 knockout mice, strongly suggesting that actin activates inflammatory cells *via* TREM-1 (55).

## Immune Responses in TREM-1 Knockout Mice

Mice genetically deficient in TREM-1 are another valuable tool to help uncover the potential benefits of modulating TREM-1 for the treatment of sepsis. To study this, multiple knockout models with different genetic modifications have been developed: TREM-1 knockout, endothelial cell-specific TREM-1 (endoTREM-1) knockout, TREM-1/3 double knockout, and TREM-like transcript-1 (TLT-1) knockout. To determine the effects of TREM-1 deficiency on the immune response to infection, TREM-1^-/-^ mice were infected with *Leishmania major* (18). Compared to WT mice, TREM-1^-/-^ mice had reduced infiltration of neutrophils and decreased lesion size around the site of bacterial inoculation (18). TREM-1^-/-^ mice also had reduced morbidity after infection with influenza A virus, measured by body weight and temperature, and decreased IL-6 in bronchoalveolar lavage (18). Importantly, while infection-related pathologies were improved, TREM-1^-/-^ mice had equivalent pathogen clearance of *L. major*, influenza virus, and *Legionella pneumophila* as WT mice, indicating these mice do not develop disseminated infection like TREM-1/3^-/-^ (18). Additionally, after LPS-induced septic shock, TREM-1^-/-^ mice had decreased neutrophil extracellular trap (NET) release in the serum and lungs, which have pro-inflammatory functions that contribute to the progression of septic shock (56). Aortas and mesenteric arteries isolated from TREM-1^-/-^ mice were protected from *in vitro* NET-induced vascular dysfunction (possibly due to their inability to respond to the eCIRP present in NETs), maintaining normal contraction and relaxation (56–58). The involvement of TREM-1 in sepsis induced vascular dysfunction was further proven using endoTREM-1^-/-^ mice (57). After cecal ligation and puncture (CLP)-induced sepsis, endoTREM-1^-/-^ mice had restored vasorelaxation in addition to reduced serum VCAM-1 and IL-6, and prolonged 7-day survival (57).

In mice, the gene for TREM-3 is adjacent to TREM-1 and likely occurred from a duplication event, demonstrated by their high homogeneity (59). Additionally, the two receptors are both amplifiers of the immune response, and likely work synergistically (59–61). TREM-3 in humans, however, is a pseudogene and has no functional overlap with TREM-3 in mice (61). Therefore, it has been suggested that TREM-1/3^-/-^ mice might better reflect TREM-1 deficiency in humans than mice deficient in TREM-1 alone (61–63). Interestingly, although TREM-1^-/-^ mice were protected after exposure to infectious agents, TREM-1/3^-/-^ mice had worse outcomes after infection. TREM-1/3^-/-^ mice had increased mortality and bacterial dissemination after infection with *Streptococcus pneumoniae* and *Klebsiella pneumoniae*, despite infected primary TREM-1/3^-/-^ macrophages having decreased cytokine release (64, 65). The relevance of murine TREM-3 to human TREM-1 function should continue to be explored to better characterize which murine models best emulate human disease.

TLT-1 belongs to the TREM family, and its gene resides in the human TREM gene cluster, along with TREM-1, -2, -3, and TLT-2 (66). It is specific to platelets and megakaryocytes and, upon platelet activation, translocates to the cell surface where it plays a role in hemostasis/thrombosis (66). Additionally, a soluble fragment of TLT-1 is present in the circulation and is believed to function as an endogenous TREM-1 inhibitor (67, 68). TLT-1 knockout mice (treml-1^-/-^) had increased gene and protein expression of inflammatory cytokines in the lungs and plasma after CLP induces sepsis (68). Additionally, septic treml-1^-/-^ mice had increased mortality, indicating a protective role of TLT-1 in polymicrobial sepsis, likely by inhibiting TREM-1 (68).

## TREM-1 Blockade as a Therapeutic Approach

Studies using TREM-1 knockout mice have demonstrated that this receptor plays a critical role in the progression of sepsis. Armed with this knowledge, multiple receptor antagonists have been created, predominately based on the structure of TREM-1 and its ligands. Modulation of TREM-1 activation with many of these inhibitors has been demonstrated as an effective approach for attenuating sepsis severity (**Table 1**).

**Table 1:** TREM-1 pathway inhibitory strategies.

Treatment	Sepsis Model	Cell/Animal	Effect	Ref.
sTREM-1	LPS	Macrophages	↓ mRNA of inflammatory cytokines	(69)
	*S. suis intraperitoneal*	Mice	↓ serum TNF-α, IL-1β, ↓ acute lung injury, ↑ survival (when given with antibiotics)	(70)
TREM-1 Fc fusion protein	LPS	Monocytes	↓ release TNF-α, IL-1β	(8)
	*P. aeruginosa*	Macrophages	↓ release TNF-α, IL-1β, MCP-1	(71)
	*S. pyogenes*	Neutrophils	↓ release IL-6, TNF-α	(72)
	LPS *intraperitoneal*	Mice	↓ serum TNF-α, IL-1β, ↓ recruitment of peritoneal macrophages and neutrophils, ↑ survival	(8)
	CLP	Mice	↑ survival	(8)
	*E. coli intraperitoneal*	Mice	↑ survival	(8)
	*S. pyogenes intravenous*	Mice	↓ serum IL-6, TNF-α, ↑ survival	(72)
	*P. aeruginosa intraperitoneal*	Mice	↓ serum IL-1β, TNF-α, MCP-1, ↑ survival	(71)
LP17	LPS	Monocytes	↓ release TNF-α, IL-1β	(73)
	*E. coli*	Neonatal leukocytes	↓ release TNF-α, IL-6, IL-8	(74)
	LPS *intraperitoneal*	Mice	↑ survival	(35)
	CLP	Mice	↓ serum TNF-α, IL-1β, ↑ survival	(73)
	*S. pyogenes intravenous*	Mice	↑ survival	(72)
	LPS *intraperitoneal*	Rats	↑ hemodynamics, ↓ serum TNF-α, IL-1β	(35)
	CLP	Rats	↑ hemodynamics, ↓ TNF-α, IL-1β, IL-6, ↑ survival	(35, 75)
	*P. aeruginosa intratracheal*	Rats	↓ serum and broncoalveolar lavage TNF-α, IL-1β, IL-6, lactic acidosis, hypoxia, ↑ hemodynamics, ↑ survival	(76)
GF9	LPS	Macrophages	↓ release TNF-α, IL-1β, IL-6	(77)
	LPS *intraperitoneal*	Mice	↓ serum TNF-α, IL-1β, IL-6, ↑ survival	(77)
SLC-TREM-1	LPS	Endothelium	↓ TREM-1 expression, ↓ MCP-1, IL-8 release	(78)
	CLP	Mice	↑ survival	(78)
M3	LPS *intraperitoneal*	Mice	↓ serum TNF-α, IL-6, ↑ survival	(41)
	CLP	Mice	↓ serum AST, ALT, TNF-α, IL-6, ↓ acute lung injury, ↑ survival	(41)
	Cecal Slurry	Neonatal Mice	↓ serum TNF-α, IL-1β, IL-6, INFγ, ↓ cardiac and pulmonary IL-1β, IL-6, ↓ cardiac dysfunction, ↑ survival	(79)
N1	LPS	Mononuclear Cells/Monocytes	↓ mRNA TNF-α, IFNγ, IL-1β, and IL-6	(80)
	LPS *intrabronchial*	Mice	↓ serum INFγ, IL-4, ↓ lung damage	(80)
LR17/LR12	LPS	Granulocyte	↓ mRNA and protein TNF-α, IL-8, IL-10, ↓ ROS production	(23)
	LPS	Monocytes	↓ release TNF-α	(23)
	LPS *intraperitoneal*	Mice	↓ serum TNF-α, IL-6, IL-10, ↑ survival	(23)
	CLP	Mice	↓ cytokines in serum, peritoneal and bronchoalveolar fluid, liver, lung, ↓ acute lung injury, ↓ bacteria in spleen, blood, ↓ TREM-1 expression, ↑ survival	(23, 57)
	Cecal Slurry	Pigs	↑ cardiac function, ↓ coagulopathy, ↓ failure of lungs, liver, kidney, ↑survival	(68)
	LPS *intravenous*	Monkeys	↑ hemodynamics, ↓ serum cytokines	(81)
Nangibotide	Sepsis	Humans	↓ systemic IL-6, ↓ SOFA scores, minimal side effects	(82, 83)

↓- decreased; ↑- increased.

### Soluble TREM-1 (sTREM-1)

**Figure 1:**
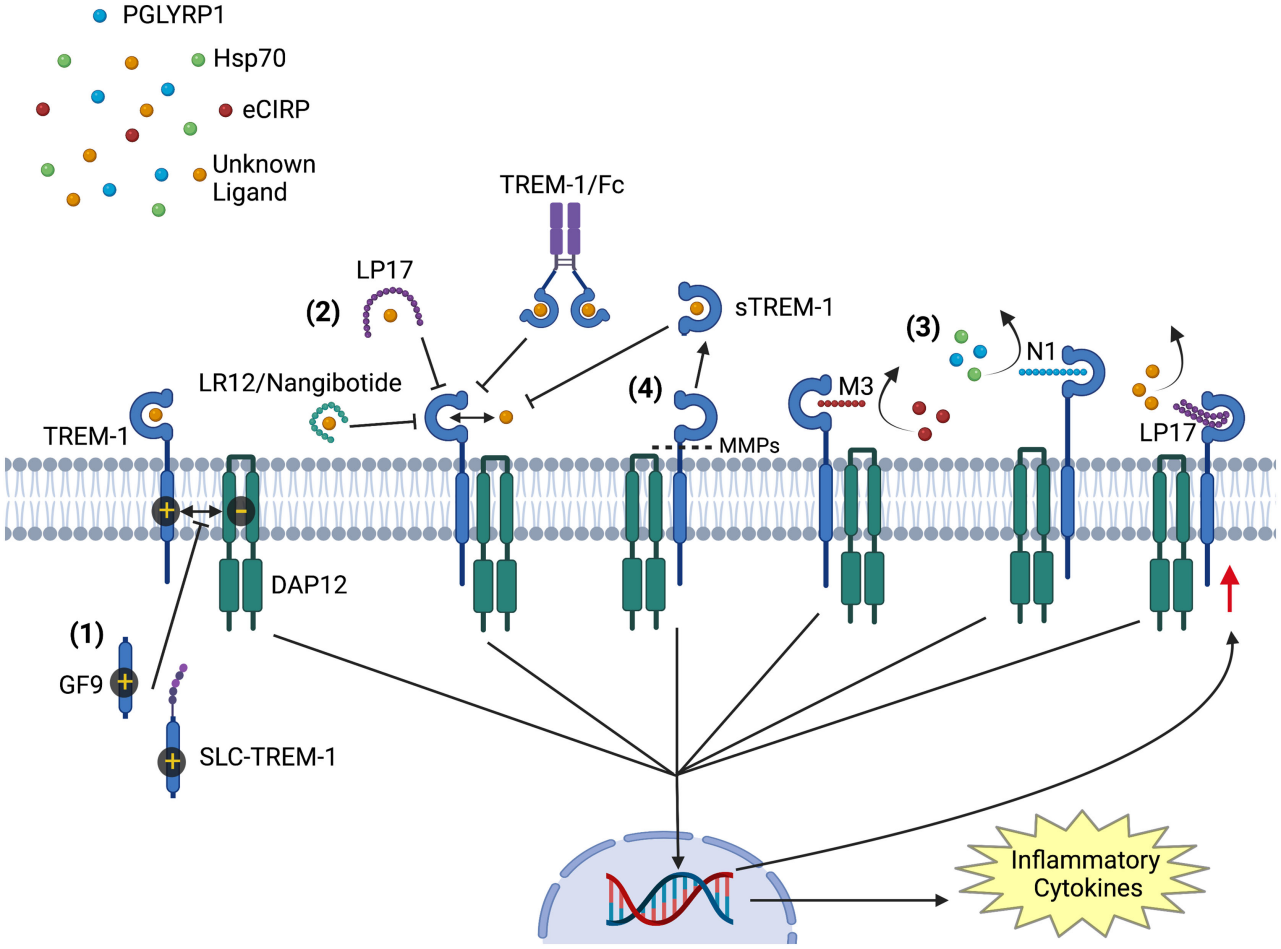
Modulators of TREM-1 Activation. Activation of TREM-1 is inhibited by multiple agents: (1) GF9 and sneaking ligand construct to TREM-1 inhibit the interaction of TREM-1 with its signaling partner DAP12. (2) LR12/nangibotide, LP17, and TREM-1/Fc fusion protein act as decoy receptors and compete for binding with naturally occurring activating ligands. (3) Together with M3 and N1, LP17 also binds to TREM-1 and competitively inhibits ligand binding. M3 competitively inhibits TREM-1 binding to and activation by extracellular cold inducible RNA binding protein, while N1 inhibits TREM-1 activation by PGLYRP1 and HSP70. (4) soluble TREM-1 is generated from proteolytic cleavage of membrane bound TREM-1 by matrix metalloproteinases. Circulating soluble TREM-1 competitively binds TREM-1’s ligands and prevents further activation. All inhibitors prevent the downstream signaling cascade that upregulates the translation of inflammatory cytokines and TREM-1 receptor. *Image created with BioRender*.

The sTREM-1 molecule is the cleaved extracellular domain of TREM-1 that was found to be increased in the blood during sepsis (69, 73, 84, 85). In addition to being studied as a prognostic indicator of sepsis severity, it has also been proposed to act as a decoy receptor for TREM-1 by binding its ligands and reducing its activation and the subsequent pro-inflammatory cytokine release (73, 86–89) (**Figure 1**). To test this hypothesis, the gene for recombinant porcine sTREM-1 was transfected into *Escherichia coli* for cloning and expression (69). After purification, it was administered to LPS-stimulated primary porcine alveolar macrophages. Macrophages treated with sTREM-1 had reduced mRNA expression of type-I and type-II inflammatory cytokines, including TNF-α, IL-1β, IL-2, IL-4, IL-8, IL-10, IL-12, IL-16, and IL-18 (69).

A similar method of genetic cloning was used to express and purify murine sTREM-1, which was named recombinant extracellular domain of TREM-1 (rTREM-1) (90). Mice were then infected with *Streptococcus suis*, a bacterium that can rapidly cause streptococcal toxic-shock-like syndrome (STSLS), followed by treatment with rTREM-1. Conversely to the *in vitro* study using porcine sTREM-1, mice inoculated with *S. suis* and treated with rTREM-1 had worse outcomes. rTREM-1 treatment was associated with increased 7-day mortality, and elevated IL1-β, TNF-α, KC, and bacterial load in both blood and peritoneal fluid (90). Interestingly, however, when rTREM-1 treatment is combined with antibiotics, mice infected with *S. suis* had better outcomes than with antibiotics alone (70). Mice that received combination treatment of rTREM-1 and ampicillin had a higher 7-day survival, lower serum levels of IL1-β and TNF-α, and attenuated acute lung injury compared to mice that received PBS vehicle, just ampicillin, or rTREM-1 alone (70). These data suggest that, while TREM-1 agonism may help clear some bacterial infections, it is also a key contributor to the detrimental inflammatory processes that culminate in acute lung injury. Many of the following TREM-1 inhibitory peptides developed as a treatment modality for sepsis are based on amino acid (aa) sequences found in sTREM-1, further demonstrating that sTREM-1 is effective as an anti-inflammatory mediator (88).

### TREM-1 Fc Fusion Protein

TREM-1/Fc, a fusion protein consisting of the extracellular domain of mouse TREM-1 and the Fc portion of human IgG1, was developed as a TREM-1 decoy receptor that can also promote the clearance of TREM-1 ligands from the circulation *via* Fc-receptor mediated endocytosis (8) (**Figure 1**). Mice treated with TREM-1/Fc prior to LPS-induced endotoxemia had decreased serum TNF-α and IL-1β and recruitment of peritoneal neutrophils and macrophages (8). Additionally, mice treated with TREM-1/Fc had improved 7-day survival in LPS, CLP, and *Escherichia coli-*induced sepsis (8). These findings were reproduced in another study using intraperitoneal administration of *Pseudomonas aeruginosa* to induce sepsis (71). Treatment with TREM-1/Fc in these mice led to improved survival and a significant reduction in serum pro-inflammatory cytokines, including TNF-α, IL-1β, INF-γ, and MCP-1 (71). Additionally, TREM-1/Fc provided protection in mice against sepsis after intravenous (i.v.) injection of *Streptococcus pyogenes* (72). Mice treated 2 hours before and 2 hours after inoculation of *S. pyogenes* had prolonged survival and decreased levels of IL-6 and TNF-α in the serum (72). In addition to its TREM-1 inhibitory effects, it is possible that the TREM-1/Fc fusion protein also enhances the phagocytic clearance of pathogens by bridging peptidoglycan-bound PGLYRP1 on the surface of bacteria with Fc-receptors on macrophages and neutrophils.

### LP17

LP17 was one of the first peptides developed to inhibit TREM-1 (73). It has a 17-aa sequence (LQVTDSGLYRCVIYHPP) derived from on a highly conserved extracellular domain of TREM-1 in both mice and humans, and is intended to operate through a mechanism analogous to that of decoy receptors (35, 73, 76). To demonstrate its intended function, flow cytometry was used to show LP17 inhibits the binding of a fluorophore conjugated-mTREM-1/IgG1 antibody to TREM-1 on murine peritoneal exudate cells (35). There is evidence that it functions both as a direct competitive inhibitor by binding to the receptor and thus preventing its activation by the ligand, and as a decoy receptor by binding TREM-1’s ligands before they are able to activate the TREM-1 receptor (4, 68) (**Figure 1**). Efficacy was initially demonstrated by reducing TNF-α and IL-1β release from LP17-treated monocytes after stimulation with LPS in a dose dependent manner (73). LP17 was further tested *in vivo* in mice subjected to either endotoxemia or CLP-induced sepsis (73). Mice treated with LP17 had decreased levels of serum TNF-α and IL-1β and improved survival after LPS injection and CLP (73). LP17 improved survival in endotoxemic mice when given 1 hour before or 4 hours after LPS injection (35, 73). Additionally, LP17 has been shown to improve survival in mice after i.v. inoculation with *Streptococcus pyogenes* (72).

LP17 has also been shown to be efficacious in treating septic rats. Rats treated with LP17 1 hour after LPS injection had attenuated sepsis severity, measured by improved hemodynamic parameters including mean arterial pressure, aortic blood flow, mesenteric blood flow, pH, and serum lactate (35). Additionally, these rats had lower concentrations of serum TNF-α, IL-1β, and nitrates/nitrites (35). Rats treated with LP17 after CLP also had improved hemodynamic parameters, serum TNF-α, IL-1β, IL-6 and nitrates/nitrites, and survival at 48 hours and 7 days (35, 75). LP17 has also been shown to be an effective treatment for sepsis secondary to *Pseudomonas aeruginosa* pneumonia in rats, resulting in with improved hemodynamic status, attenuated lactic acidosis and hypoxemia, reduced serum TNF-α, IL-1β, and IL-6, and improved 7-day survival of the septic rats (76).

In addition to the studies in adult rodents, LP17 has been considered as a potential therapeutic agent for neonatal sepsis (74). In an *in vitro* study, leukocytes isolated from umbilical cord blood from full term human neonates and treated with LP17 had decreased production of TNF-α, IL-6, and IL-8 after exposure to *Escherichia coli* (74).

### M3

M3 is another inhibitory peptide of TREM-1 that was developed to specifically inhibit activation by its novel ligand, eCIRP (41). Its 7-aa sequence (RGFFRGG) was designed based on an area of homology between PGLYRP1 and CIRP (30, 41). Using a FRET assay, M3 was shown to dramatically abrogate CIRP’s binding to TREM-1 in both murine peritoneal macrophages and RAW264.7 cells (41) (**Figure 1**). To test this peptide *in vivo*, mice were subjected to LPS-endotoxemia and those treated with M3 had decreased levels of serum TNF-α and IL-6, and improved 7-day survival (41). M3 was also protective in mice with CLP-sepsis, measured by decreased levels of serum AST, ALT, TNF-α and IL-6, and attenuated severity of sepsis-associated acute lung injury (41, 91). Mice treated with intraperitoneal (i.p.) M3 at the time of CLP or 90 minutes later also showed a 10-day survival benefit (41).

Like LP17, M3 has also been shown to be effective in treating sepsis in neonates (79). Using a cecal slurry model of sepsis, neonatal mice treated with i.p. M3 had attenuated disease severity, as measured by a reduction in serum IL-6, TNF-α, IL-1β, and IFN-γ, and improved pulmonary and cardiac inflammation (79). Additionally, M3-treated neonates had improved cardiac function, measured by cardiac output and left-ventricular end diastolic diameter. Importantly, neonatal mice treated with M3 at either the time of cecal slurry injection or 2 hours later had improved 7-day survival (79). Although M3 is a novel peptide requires further studies, it shows promise in treating sepsis in both neonates and adults.

### N1

N1 is a 10-aa inhibitory peptide based on the N-terminal aa sequence (aa 77–86) of PGLYRP1 (80). Using affinity chromatography, N1 was shown to bind sTREM-1 immobilized on CNBr-activated Sepharose (80). Using immunoblotting, N1 was also shown to bind TREM-1 on the surface of monocytes (80) (**Figure 1**). Monocytes and lymphocytes exposed to PGLYRP1 and Hsp70, two previously mentioned ligands of TREM-1, had decreased LDH release in the presence of N1. Additionally, N1 treated cells had decreased mRNA expression of TNF-α, IFNγ, IL-1β, and IL-6 after exposure to LPS (80). In mice with acute lung injury after bronchial instillation of LPS and α-galactosylceramide, i.v. treatment with N1 protected against the resultant cytokine storm, leading to decreased serum levels of INFγ and IL-4. These mice also had reduced histologically evaluated pulmonary inflammation (80). N1 is another novel peptide that effectively inhibits TREM-1 induced inflammation, but requires additional studies looking at its efficacy in a polymicrobial model of sepsis to better evaluate it as a treatment modality.

### GF9

Moving away from the strategy of preventing ligands from interacting with TREM-1, GF9 is a ligand-independent peptide (GLLSKSLVF) derived from murine TREM-1’s transmembrane region, designed using the Signaling Chain HOmoOligomerization (SCHOOL) model (92). It functions by inhibiting the interaction between TREM-1 and its signaling partner, DAP12 (77, 92) (**Figure 1**). Using a mouse model of endotoxemia, the efficacy of i.p. GF9 was studied, administered in its free form, and carried within high density lipoproteins (HDL). Synthetic HDL was modified to target delivery and uptake by macrophages, therefore delivering the GF9 directly to the cell of interest. Mice subjected to endotoxemia had higher survival after pretreatment with GF9 both in free form and incorporated into macrophage-targeted HDL-like particles. However, mice required a dose of 25mg/kg of free GF9 to see a survival benefit compared to vehicle, where a significantly lower dose of 5mg/kg of HDL bound GF9 was able to achieve the same survival benefit. Additionally, incorporation of GF9 into HDL prolonged the peptide’s half-life, which together with its targeted macrophage delivery could contribute to its reduced effective dose (77).

### SLC-TREM-1

TREM-1 sneaking ligand construct (SLC-TREM-1) is another protein that inhibits the interaction of TREM-1 with DAP12 (**Figure 1**), but it was specifically designed to target the endothelium (78). It is composed of 3 portions: an E-selectin targeting domain that binds to the surface of endothelial cells, *Pseudomonas aeruginosa* exotoxin A to facilitate translocation from the endosomal vesicular system into the cytosol, and a 7-aa sequence (LSKSLVF) derived from the transmembrane region of TREM-1, and actually contained within GF9 (77, 78, 93). Endothelial cells stimulated with LPS *in vitro* had decreased TREM-1 expression and activation after treatment with SLC-TREM-1 (78). Additionally, treatment with this protein intraperitoneally improved the 10-day survival in mice subjected to CLP (78). GF9 and SLC-TREM-1 demonstrate that targeting the interaction of TREM-1 with DAP12 is a viable TREM-1 inhibitory strategy with potential for drug development in sepsis.

### LR17/LR12

LR17/LR12 are the most studied inhibitory peptides of TREM-1. LR-17 is a 17-aa peptide (LQEEDAGEYGCMVDGAR) based on a highly conserved sequence between TREM-1 and TREM-like transcript-1 (TLT-1), a membrane bound protein found on the surface of activated platelets (23, 68, 94) (**Figure 1**). After demonstrating structural similarities between TREM-1 and TLT-1 using crystallographic approaches, soluble TLT-1 (sTLT-1) was studied as a modulator of the inflammatory response and shown to decrease human neutrophil production of TNF-a, IL-6, and IL-8 after LPS stimulation (23, 94). LR17 was subsequently shown to protect mice from LPS endotoxemia (23). Mice treated with LR17 before or after LPS administration had improved 10-day survival and decreased serum pro-inflammatory cytokines (23). This peptide also has a protective effect in mice with CLP-sepsis (23). Mice treated with LR17 had decreased sepsis severity demonstrated by lower levels of IL-6, TNF-a, and IL-10 in serum, peritoneal and bronchoalveolar fluid, and IL-6 and TNF-a in the liver, and lung (23). LP17 also ameliorated sepsis-induced acute lung injury, reduced bacterial load in the spleen and blood, and an improved 7-day survival (23).

It was discovered that just 12 aa accounted for the LP17’s anti-inflammatory effects, and a novel peptide containing this sequence (LQEEDAGEYGCM) was created and named LR12 (4, 68). After confirming TREM-1’s expression on vascular endothelial cells, the effects of LR12 on vascular reactivity during sepsis was explored (57). Using both CLP-sepsis and LPS endotoxemia, it was determined that LR12 prevents the TREM-1 upregulation induced in the murine aortas and mesenteric arteries of mice subjected to these models (57). Additionally, LR12 treatment protected mice from sepsis-induced vascular dysfunction, measured by improved vascular contractility, and attenuated IL-6, TNF-α, and IL-10 expression as well as NOS and COX signaling pathway activation in murine aortas and mesenteric arteries (57).

The effects of LR12 during sepsis were further studied in adult minipigs to better characterize its beneficial properties as a therapeutic agent (68). Polymicrobial sepsis was induced using a cecal slurry model. Pigs treated with LR12 had less cardiovascular failure, measured by MAP, vasopressor use, and cardiac index (68). Additionally, they had decreased coagulopathy and organ failure in the lungs, liver, and kidney. LR12 improved 24-hour survival in septic pigs from 40% to 100% (68). The effects of LR12 was further explored in nonhuman primates using an LPS model of endotoxemia (81). Monkeys received an i.v. bolus of LPS followed by a continuous infusion of LR12 or placebo for 8 hours. Monkeys treated with LR12 were protected from hypotension and neutropenia and had reduced plasma cytokine concentrations (81). One month after LR12 administration, no side effects were noted in the treated animals (81). In addition to i.v. infusion, a sustained release implant was tested as a novel administration route for LR12 in rats (95). An *in situ* poly-lactide-co-glycolide (PGLA) or poly-lactide (PLA) implant was used to deliver LR12 in a dimerized formulation to diminish peptide degradation and allow sustained release (95). In healthy rats, these *in situ* forming implants delivered a therapeutic concentration of LR12 for 7 days, introducing an effective method of administrating this otherwise short-lived peptide (95).

### Nangibotide

LR12 is the first TREM-1 inhibitor to have reached the clinical stage. It is currently being studied in Phase 2 clinical trials of sepsis, COVID-19, and acute myocardial infarction under the name Inotrem (nangibotide) (16, 82, 83, 96–98). Nangibotide is an immune modulator targeting the inflammatory response amplification maintained by TREM-1 (82). In Phase 1 trials, continuous i.v. administration of nangibotide was safe and well tolerated at doses up 6 mg/kg/h for 7 hours and 45 mins following a 15-minute loading dose of 5 mg/kg, with few adverse events noted after 28 days of followup (83). In a Phase 2a multicenter, randomized, double-blind, placebo-controlled clinical trial, patients received a continuous infusion of 0.3, 1.0, or 3.0 mg/kg/h of nangibotide within 24 hours of the diagnosis of septic shock (82). Treatment was continued until the patient was off vasopressors for 12 hours, or for a maximum of 5 days. There was no difference in adverse events, tolerability, inflammatory biomarkers and clinical efficacy between study groups (82). Treatment with nangibotide, however, was associated with a decrease in the serum levels of IL-6 (82). In a subgroup analysis, patients with high sTREM-1 treated with nangibotide had decreased SOFA scores after treatment (82, 98), suggesting that sTREM-1 could be a potential biomarker to predict which septic patients respond to nangibotide. A pivotal Phase 2b trial, looking at the efficacy, safety, and tolerability of nangibotide in patients with septic shock is currently underway (16, 97). Patients are given two doses of the peptide for 3-5 days, depending on vasopressor requirements. The primary endpoint being evaluated is total SOFA score changes from baseline to day 5 in all patients, and will include a subgroup analysis of patients with elevated sTREM-1 baseline levels (16). Larger studies are still needed to investigate the efficacy of nangibotide in attenuating sepsis severity, and to evaluate sTREM-1 as a potential biomarker of patient response to nangibotide.

## Summary and Perspectives

Over the last two decades, extensive research has revealed TREM-1’s critical role as a mediator of inflammation in sepsis and other diseases. Substantial work has been done to identify its endogenous ligands and develop inhibitors to be used as potential therapeutic agents for a range of diseases. There are now a variety of inhibitory molecules, predominately oligopeptides, that have shown promise as a treatment for sepsis using various preclinical sepsis models in a number of species. These peptides are capable of targeting TREM-1 activation at various points within its signaling cascade, from acting as a decoy receptor and binding up its endogenous ligands, to binding the extracellular domain and competitively inhibiting the binding of endogenous ligands, to interrupting the transmembrane association of TREM-1 with DAP12. Beyond oligopeptides, other modulators of the TREM-1 pathway have been studied in non-infectious inflammatory diseases (99). Researchers in Sweden have developed an anti-TREM-1 antibody that reduces secretion of proinflammatory cytokines from lamina propria cells isolated from patients with inflammatory bowel disease (99). Targeted monoclonal antibody treatment has been effective in treating a variety of inflammatory diseases, and TREM-1 targeted antibodies may be a beneficial approach to sepsis drug development. Future research could benefit from investigating this novel anti-TREM-1 antibody in septic preclinical models.

Although many TREM-1 inhibitory peptides have shown promise in preclinical models, there are currently no FDA approved treatments for sepsis. Strategies targeting inflammatory mediators like TNF-α and IL-1β were not effective in clinical trials for sepsis, despite efficacy in experimental models (100). Further, drotrecogin alpha, the only treatment to receive FDA approval for the treatment of sepsis was later withdrawn from the market (101). This recombinant human activated protein C did not reduce mortality in septic patients at 28 days in a follow-up phase 3 international, randomized, controlled trial, and is no longer approved as a treatment for sepsis (101). There are likely many barriers to creating effective sepsis treatments, including poor translation between animal models and human disease, heterogeneity of the patient population selected for clinical trials, and the overall heterogeneity of the etiology of the disease (102). The physiologic response to sepsis in mice differs from humans, and mice are also more resistant to endotoxin and resilient to infection. Further, many treatments tested in murine models are given before, during, or shortly after infectious exposure. Conversely, patients often present for treatment days after infection, with sepsis developing over time. Additionally, in preclinical models, treatments are often tested on sepsis from a single, well-defined source of infection, where clinical trials often include patients who present with sepsis from a wide variety of etiologies. The host response has great variation based on the location of infectious insult and inciting organism species. Patients also have a variety of specific characteristics, like sex, age, comorbidities, and medications that can all effect response to an experimental treatment. In that regard, clinical trials of sepsis treatments would possibly benefit from selecting patients according to the source of infection and infectious agent.

LR12, a 12-aa peptide, has been studied extensively in mice, rats, pigs, and nonhuman primates as a treatment for sepsis and has been shown to be effective and safe when administered as an i.v. bolus, continuous infusion, and an *in situ* forming implant. It is currently in phase 2b clinical trials under the name nangibotide and has so far been demonstrated to be safe and apparently effective in Phase-1 and Phase-2a studies. This peptide shows significant promise as a treatment for septic patients. It has demonstrated efficacy in seven preclinical models and in four different species, and has been administered up to 24 hours after onset of infection, addressing key issues of translatability and time of treatment in patients. Further, in the current trial, investigators are identifying patients with elevated levels of plasma sTREM-1 to evaluate whether these patients are better responder and, consequently, are more likely to benefit from treatment. By identifying a subgroup of patients who best respond to this treatment, administration can be personalized and efficacy can be better assessed. Sepsis has been called a “graveyard for pharmaceutical companies”. Targeting the TREM-1 activation pathway, however, shows great promise and may finally become the first safe and effective therapeutic strategy to treat septic patients.

## Author Contributions

SS and MB conceived the original idea. SS prepared the figures and wrote the manuscript. PW and MB revised and edited the manuscript. PW supervised the project. All authors contributed to the article and approved the submitted version.

## Funding

This study was partially supported by the National Institutes of Health (NIH) grants R35GM118337 and U01AI33655.

## Conflict of Interest

The authors declare that the research was conducted in the absence of any commercial or financial relationships that could be construed as a potential conflict of interest.

## Publisher’s Note

All claims expressed in this article are solely those of the authors and do not necessarily represent those of their affiliated organizations, or those of the publisher, the editors and the reviewers. Any product that may be evaluated in this article, or claim that may be made by its manufacturer, is not guaranteed or endorsed by the publisher.

## Abbreviations

aa, Amino acid; AKT, Protein kinase B; ALI, Acute lung injury; ALT, Alanine aminotransferase; AST, Aspartate aminotransferase; BAL, Bronchoalveolar lavage; BMDN, Bone marrow-derived neutrophils; CIRP, Cold-inducible RNA-binding protein; CLP, Cecal ligation and puncture; DAMP, Damage-associated molecular pattern; DAP12, DNAX activation protein 12; eCIRP, Extracellular cold-inducible RNA-binding protein; ERK, Extracellular signal-regulated kinase; FRET, Fluorescence resonance energy transfer; HDL, High density lipoprotein; HMGB1, High mobility group box 1; HSP70, 70 kDa heat shock protein; IFN, Interferon; IgG, Immunoglobulin G; IκBα, Nuclear factor of kappa light polypeptide gene enhancer in B-cells inhibitor alpha; IL, Interleukin; IP, Intraperitoneal; ITAM, Immunoreceptor tyrosine-based activation motif; IV, Intravenous; KO, Knockout; LDH, Lactate dehydrogenase; LPS, Lipopolysaccharides; MAP, Mean arterial pressure; MAPK, Mitogen-activated protein kinase; MCP, Macrophage chemoattractant protein; MMP, Metalloproteinases; mRNA, Messenger ribonucleic acid; MyD88, Myeloid differentiation primary response 88; NET, Neutrophil extracellular trap; NF-κB, Nuclear factor kappa-light-chain-enhancer of activated B cells; PAMPs, Pathogen-associated molecular patterns; PBMC, Peripheral blood mononuclear cell; PBS, Phosphate buffered saline; PGLA, Poly-lactide-co-glycolide; PGLYRP1, Peptidoglycan recognition protein 1; PGN, Peptidoglycan; PI3K, Phosphatidylinositol 3-kinase; PLA, Poly-lactide; PMN, Polymorphonuclear leukocyte; PRR, Pattern recognition receptor; RAGE, Receptor for advanced glycation end products; RAS, Reticular activating system; rm, Recombinant murine; SCHOOL, Signaling chain homooligomerization; SLC-TREM-1, Triggering receptor expressed on myeloid cells-1 sneaking ligand construct; SPR, Surface plasmon resonance; sTREM-1, Soluble TREM-1; STSLS, Streptococcal toxic-shock-like syndrome; Syk, spleen tyrosine kinase; TLR, Toll-like receptor; TLT-1, Triggering receptor expressed on myeloid cells-1-like transcript 1; TNF-α, Tumor necrosis factor-α; TREM-1, Triggering receptor expressed on myeloid cells-1; WT, Wild-type.

## Glossary

**Table:**

aa	Amino acid
AKT	Protein kinase B
ALI	Acute lung injury
ALT	Alanine aminotransferase
AST	Aspartate aminotransferase
BAL	Bronchoalveolar lavage
BMDN	Bone marrow-derived neutrophils
CIRP	Cold-inducible RNA-binding protein
CLP	Cecal ligation and puncture
DAMP	Damage-associated molecular pattern
DAP12	DNAX activation protein 12
eCIRP	Extracellular cold-inducible RNA-binding protein
ERK	Extracellular signal-regulated kinase
FRET	Fluorescence resonance energy transfer
HDL	High density lipoprotein
HMGB1	High mobility group box 1
HSP70	70 kDa heat shock protein
IFN	Interferon
IgG	Immunoglobulin G
IκBa	Nuclear factor of kappa light polypeptide gene enhancer in B-cells inhibitor alpha
IL	Interleukin
IP	Intraperitoneal
ITAM	Immunoreceptor tyrosine-based activation motif
IV	Intravenous
KO	Knockout
LDH	Lactate dehydrogenase
LPS	Lipopolysaccharides
MAP	Mean arterial pressure
MAPK	Mitogen-activated protein kinase
MCP	Macrophage chemoattractant protein
MMP	Metalloproteinases
mRNA	Messenger ribonucleic acid
MyD88	Myeloid differentiation primary response 88
NET	Neutrophil extracellular trap
NF-κB	Nuclear factor kappa-light-chain-enhancer of activated B cells
PAMPs	Pathogen-associated molecular patterns
PBMC	Peripheral blood mononuclear cell
PBS	Phosphate buffered saline
PGLA	Poly-lactide-co-glycolide
PGLYRP1	Peptidoglycan recognition protein 1
PGN	Peptidoglycan
PI3K	Phosphatidylinositol 3-kinase
PLA	Poly-lactide
PMN	Polymorphonuclear leukocyte
PRR	Pattern recognition receptor
RAGE	Receptor for advanced glycation end products
RAS	Reticular activating system
rm	Recombinant murine
SCHOOL	Signaling chain homooligomerization
SLC-TREM-1	Triggering receptor expressed on myeloid cells-1 sneaking ligand construct
SPR	Surface plasmon resonance
sTREM-1	Soluble TREM-1
STSLS	Streptococcal toxic-shock-like syndrome
Syk	spleen tyrosine kinase
TLR	Toll-like receptor
TLT-1	Triggering receptor expressed on myeloid cells-1-like transcript 1
TNF-α	Tumor necrosis factor-α
TREM-1	Triggering receptor expressed on myeloid cells-1
WT	Wild-type.

## References

[1] Hotchkiss RS Moldawer LL Opal SM Reinhart K Turnbull IR Vincent JL. Sepsis and Septic Shock. Nat Rev Dis Primers (2016) 2:16045. doi: 10.1038/nrdp.2016.45 28117397 PMC5538252

[2] van der Poll T van de Veerdonk FL Scicluna BP Netea MG. The Immunopathology of Sepsis and Potential Therapeutic Targets. Nat Rev Immunol (2017) 17(7):407–20. doi: 10.1038/nri.2017.36 28436424

[3] Amarante-Mendes GP Adjemian S Branco LM Zanetti LC Weinlich R Bortoluci KR. Pattern Recognition Receptors and the Host Cell Death Molecular Machinery. Front Immunol (2018) 9:2379. doi: 10.3389/fimmu.2018.02379 30459758 PMC6232773

[4] Dantas PHDS Matos AO da Silva Filho E Silva-Sales M Sales-Campos H. Triggering Receptor Expressed on Myeloid Cells-1 (TREM-1) as a Therapeutic Target in Infectious and Noninfectious Disease: A Critical Review. Int Rev Immunol (2020) 39(4):188–202. doi: 10.1080/08830185.2020.1762597 32379561

[5] Tammaro A Derive M Gibot S Leemans JC Florquin S Dessing MC. TREM-1 and Its Potential Ligands in Non-Infectious Diseases: From Biology to Clinical Perspectives. Pharmacol Ther (2017) 177:81–95. doi: 10.1016/j.pharmthera.2017.02.043 28245991

[6] Schiechl G Brunner SM Kesselring R Martin M Ruemmele P Mack M. Inhibition of Innate Co-Receptor TREM-1 Signaling Reduces CD4(+) T Cell Activation and Prolongs Cardiac Allograft Survival. Am J Transplant (2013) 13(5):1168–80. doi: 10.1111/ajt.12186 23463907

[7] Roe K Gibot S Verma S. Triggering Receptor Expressed on Myeloid Cells-1 (TREM-1): A New Player in Antiviral Immunity? Front Microbiol (2014) 5:627. doi: 10.3389/fmicb.2014.00627 25505454 PMC4244588

[8] Bouchon A Facchetti F Weigand MA Colonna M. TREM-1 Amplifies Inflammation and Is a Crucial Mediator of Septic Shock. Nature (2001) 410(6832):1103–7. doi: 10.1038/35074114 11323674

[9] Bouchon A Dietrich J Colonna M. Cutting Edge: Inflammatory Responses Can be Triggered by TREM-1, a Novel Receptor Expressed on Neutrophils and Monocytes. J Immunol (2000) 164(10):4991–5. doi: 10.4049/jimmunol.164.10.4991 10799849

[10] Zamyatina A Heine H. Lipopolysaccharide Recognition in the Crossroads of TLR4 and Caspase-4/11 Mediated Inflammatory Pathways. Front Immunol (2020) 11:585146. doi: 10.3389/fimmu.2020.585146 33329561 PMC7732686

[11] Cohen J. TREM-1 in Sepsis. Lancet (2001) 358(9284):776–8. doi: 10.1016/S0140-6736(01)06007-X 11564478

[12] Klesney-Tait J Turnbull IR Colonna M. The TREM Receptor Family and Signal Integration. Nat Immunol (2006) 7(12):1266–73. doi: 10.1038/ni1411 17110943

[13] Turnbull IR Gilfillan S Cella M Aoshi T Miller M Piccio L. Cutting Edge: TREM-2 Attenuates Macrophage Activation. J Immunol (2006) 177(6):3520–4. doi: 10.4049/jimmunol.177.6.3520 16951310

[14] Gibot S. Clinical Review: Role of Triggering Receptor Expressed on Myeloid Cells-1 During Sepsis. Crit Care (2005) 9(5):485–9. doi: 10.1186/cc3732 PMC1297602 16277737

[15] Gibot S Le Renard PE Bollaert PE Kolopp-Sarda MN Béné MC Faure GC. Surface Triggering Receptor Expressed on Myeloid Cells 1 Expression Patterns in Septic Shock. Intensive Care Med (2005) 31(4):594–7. doi: 10.1007/s00134-005-2572-x 15754199

[16] Francois B Lambden S Gibot S Derive M Olivier A Cuvier V. Rationale and Protocol for the Efficacy, Safety and Tolerability of Nangibotide in Patients With Septic Shock (ASTONISH) Phase IIb Randomised Controlled Trial. BMJ Open (2021) 11(7):e042921. doi: 10.1136/bmjopen-2020-042921 PMC8264912 34233965

[17] Brenner T Uhle F Fleming T Wieland M Schmoch T Schmitt F. Soluble TREM-1 as a Diagnostic and Prognostic Biomarker in Patients With Septic Shock: An Observational Clinical Study. Biomarkers (2017) 22(1):63–9. doi: 10.1080/1354750X.2016.1204005 27319606

[18] Weber B Schuster S Zysset D Rihs S Dickgreber N Schürch C. TREM-1 Deficiency can Attenuate Disease Severity Without Affecting Pathogen Clearance. PloS Pathog (2014) 10(1):e1003900. doi: 10.1371/journal.ppat.1003900 24453980 PMC3894224

[19] Colonna M Facchetti F. TREM-1 (Triggering Receptor Expressed on Myeloid Cells): A New Player in Acute Inflammatory Responses. J Infect Dis (2003) 187 Suppl 2:S397–401. doi: 10.1086/374754 12792857

[20] Arts RJ Joosten LA van der Meer JW Netea MG. TREM-1: Intracellular Signaling Pathways and Interaction With Pattern Recognition Receptors. J Leukoc Biol (2013) 93(2):209–15. doi: 10.1189/jlb.0312145 23108097

[21] Sharif O Knapp S. From Expression to Signaling: Roles of TREM-1 and TREM-2 in Innate Immunity and Bacterial Infection. Immunobiology (2008) 213(9-10):701–13. doi: 10.1016/j.imbio.2008.07.008 18926286

[22] Dower K Ellis DK Saraf K Jelinsky SA Lin LL. Innate Immune Responses to TREM-1 Activation: Overlap, Divergence, and Positive and Negative Cross-Talk With Bacterial Lipopolysaccharide. J Immunol (2008) 180(5):3520–34. doi: 10.4049/jimmunol.180.5.3520 18292579

[23] Derive M Bouazza Y Sennoun N Marchionni S Quigley L Washington V. Soluble TREM-Like Transcript-1 Regulates Leukocyte Activation and Controls Microbial Sepsis. J Immunol (2012) 188(11):5585–92. doi: 10.4049/jimmunol.1102674 PMC6382278 22551551

[24] Ford JW McVicar DW. TREM and TREM-Like Receptors in Inflammation and Disease. Curr Opin Immunol (2009) 21(1):38–46. doi: 10.1016/j.coi.2009.01.009 19230638 PMC2723941

[25] Fortin CF Lesur O Fulop T. Effects of TREM-1 Activation in Human Neutrophils: Activation of Signaling Pathways, Recruitment Into Lipid Rafts and Association With TLR4. Int Immunol (2007) 19(1):41–50. doi: 10.1093/intimm/dxl119 17098818

[26] Klesney-Tait J Colonna M. Uncovering the TREM-1-TLR Connection. Am J Physiol Lung Cell Mol Physiol (2007) 293(6):L1374–6. doi: 10.1152/ajplung.00415.2007 17934061

[27] Bleharski JR Kiessler V Buonsanti C Sieling PA Stenger S Colonna M. A Role for Triggering Receptor Expressed on Myeloid Cells-1 in Host Defense During the Early-Induced and Adaptive Phases of the Immune Response. J Immunol (2003) 170(7):3812–8. doi: 10.4049/jimmunol.170.7.3812 12646648

[28] Nathan C Ding A. TREM-1: A New Regulator of Innate Immunity in Sepsis Syndrome. Nat Med (2001) 7(5):530–2. doi: 10.1038/87846 11329047

[29] Ornatowska M Azim AC Wang X Christman JW Xiao L Joo M. Functional Genomics of Silencing TREM-1 on TLR4 Signaling in Macrophages. Am J Physiol Lung Cell Mol Physiol (2007) 293(6):L1377–84. doi: 10.1152/ajplung.00140.2007 PMC3969455 17905855

[30] Read CB Kuijper JL Hjorth SA Heipel MD Tang X Fleetwood AJ. Cutting Edge: Identification of Neutrophil PGLYRP1 as a Ligand for TREM-1. J Immunol (2015) 194(4):1417–21. doi: 10.4049/jimmunol.1402303 PMC4319313 25595774

[31] Inanc N Mumcu G Can M Yay M Silbereisen A Manoil D. Elevated Serum TREM-1 Is Associated With Periodontitis and Disease Activity in Rheumatoid Arthritis. Sci Rep (2021) 11(1):2888. doi: 10.1038/s41598-021-82335-9 33536478 PMC7859204

[32] Zulfiqar F Hozo I Rangarajan S Mariuzza RA Dziarski R Gupta D. Genetic Association of Peptidoglycan Recognition Protein Variants With Inflammatory Bowel Disease. PloS One (2013) 8(6):e67393. doi: 10.1371/journal.pone.0067393 23840689 PMC3686734

[33] Singh H Rai V Nooti SK Agrawal DK. Novel Ligands and Modulators of Triggering Receptor Expressed on Myeloid Cells Receptor Family: 2015-2020 Updates. Expert Opin Ther Pat (2021) 31(6):549–61. doi: 10.1080/13543776.2021.1883587 PMC8169545 33507843

[34] Kashyap DR Wang M Liu LH Boons GJ Gupta D Dziarski R. Peptidoglycan Recognition Proteins Kill Bacteria by Activating Protein-Sensing Two-Component Systems. Nat Med (2011) 17(6):676–83. doi: 10.1038/nm.2357 PMC3176504 21602801

[35] Gibot S Buonsanti C Massin F Romano M Kolopp-Sarda MN Benigni F. Modulation of the Triggering Receptor Expressed on the Myeloid Cell Type 1 Pathway in Murine Septic Shock. Infect Immun (2006) 74(5):2823–30. doi: 10.1128/IAI.74.5.2823-2830.2006 PMC1459741 16622220

[36] Sharapova TN Romanova EA Ivanova OK Sashchenko LP Yashin DV. Cytokines TNFα, FN and IL-2 Are Responsible for Signal Transmission From the Innate Immunity Protein Tag7 (PGLYRP1) to Cytotoxic Effector Lymphocytes. Cells (2020) 9(12):2602. doi: 10.3390/cells9122602 PMC7761954 33291689

[37] Sharapova TN Ivanova OK Soshnikova NV Romanova EA Sashchenko LP Yashin DV. Innate Immunity Protein Tag7 Induces 3 Distinct Populations of Cytotoxic Cells That Use Different Mechanisms to Exhibit Their Antitumor Activity on Human Leukocyte Antigen-Deficient Cancer Cells. J Innate Immun (2017) 9(6):598–608. doi: 10.1159/000479382 28977785

[38] Qiang X Yang WL Wu R Zhou M Jacob A Dong W. Cold-Inducible RNA-Binding Protein (CIRP) Triggers Inflammatory Responses in Hemorrhagic Shock and Sepsis. Nat Med (2013) 19(11):1489–95. doi: 10.1038/nm.3368 PMC3826915 24097189

[39] Zhang F Brenner M Yang WL Wang P. A Cold-Inducible RNA-Binding Protein (CIRP)-Derived Peptide Attenuates Inflammation and Organ Injury in Septic Mice. Sci Rep (2018) 8(1):3052–62. doi: 10.1038/s41598-017-13139-z PMC5809586 29434211

[40] Yang WL Sharma A Wang Z Li Z Fan J Wang P. Cold-Inducible RNA-Binding Protein Causes Endothelial Dysfunction *via* Activation of Nlrp3 Inflammasome. Sci Rep (2016) 6:26571. doi: 10.1038/srep26571 27217302 PMC4877585

[41] Denning NL Aziz M Murao A Gurien SD Ochani M Prince JM. Extracellular CIRP as an Endogenous TREM-1 Ligand to Fuel Inflammation in Sepsis. JCI Insight (2020) 5(5):e134172. doi: 10.1172/jci.insight.134172 PMC7141396 32027618

[42] Wu J Li J Salcedo R Mivechi NF Trinchieri G Horuzsko A. The Proinflammatory Myeloid Cell Receptor TREM-1 Controls Kupffer Cell Activation and Development of Hepatocellular Carcinoma. Cancer Res (2012) 72(16):3977–86. doi: 10.1158/0008-5472.CAN-12-0938 PMC3694446 22719066

[43] Yang H Antoine DJ Andersson U Tracey KJ. The Many Faces of HMGB1: Molecular Structure-Functional Activity in Inflammation, Apoptosis, and Chemotaxis. J Leukoc Biol (2013) 93(6):865–73. doi: 10.1189/jlb.1212662 PMC4051189 23446148

[44] Yang H Tracey KJ. Targeting HMGB1 in Inflammation. Biochim Biophys Acta (2010) 1799(1-2):149–56. doi: 10.1016/j.bbagrm.2009.11.019 PMC4533842 19948257

[45] Weigand MA Hörner C Bardenheuer HJ Bouchon A. The Systemic Inflammatory Response Syndrome. Best Pract Res Clin Anaesthesiol (2004) 18(3):455–75. doi: 10.1016/j.bpa.2003.12.005 15212339

[46] Wang X Xiang L Li H Chen P Feng Y Zhang J. The Role of HMGB1 Signaling Pathway in the Development and Progression of Hepatocellular Carcinoma: A Review. Int J Mol Sci (2015) 16(9):22527–40. doi: 10.3390/ijms160922527 PMC4613322 26393575

[47] El Mezayen R El Gazzar M Seeds MC McCall CE Dreskin SC Nicolls MR. Endogenous Signals Released From Necrotic Cells Augment Inflammatory Responses to Bacterial Endotoxin. Immunol Lett (2007) 111(1):36–44. doi: 10.1016/j.imlet.2007.04.011 17568691 PMC3034364

[48] Zhong WJ Duan JX Liu T Yang HH Guan XX Zhang CY. Activation of NLRP3 Inflammasome Up-Regulates TREM-1 Expression in Murine Macrophages *via* HMGB1 and IL-18. Int Immunopharmacol (2020) 89(Pt A):107045. doi: 10.1016/j.intimp.2020.107045 33045564 PMC7545267

[49] Lo TH Tseng KY Tsao WS Yang CY Hsieh SL Chiu AW. TREM-1 Regulates Macrophage Polarization in Ureteral Obstruction. Kidney Int (2014) 86(6):1174–86. doi: 10.1038/ki.2014.205 24918157

[50] Rosenzweig R Nillegoda NB Mayer MP Bukau B. The Hsp70 Chaperone Network. Nat Rev Mol Cell Biol (2019) 20(11):665–80. doi: 10.1038/s41580-019-0133-3 31253954

[51] Asea A Kraeft SK Kurt-Jones EA Stevenson MA Chen LB Finberg RW. HSP70 Stimulates Cytokine Production Through a CD14-Dependant Pathway, Demonstrating its Dual Role as a Chaperone and Cytokine. Nat Med (2000) 6(4):435–42. doi: 10.1038/74697 10742151

[52] Sharapova TN Romanova EA Ivanova OK Yashin DV Sashchenko LP. Hsp70 Interacts With the TREM-1 Receptor Expressed on Monocytes and Thereby Stimulates Generation of Cytotoxic Lymphocytes Active Against MHC-Negative Tumor Cells. Int J Mol Sci (2021) 22(13):6889. doi: 10.3390/ijms22136889 34206968 PMC8267615

[53] Lee WM Galbraith RM. The Extracellular Actin-Scavenger System and Actin Toxicity. N Engl J Med (1992) 326(20):1335–41. doi: 10.1056/NEJM199205143262006. 1314333

[54] Haselmayer P Grosse-Hovest L von Landenberg P Schild H Radsak MP. TREM-1 Ligand Expression on Platelets Enhances Neutrophil Activation. Blood (2007) 110(3):1029–35. doi: 10.1182/blood-2007-01-069195 17452516

[55] Fu L Han L Xie C Li W Lin L Pan S. Identification of Extracellular Actin As a Ligand for Triggering Receptor Expressed on Myeloid Cells-1 Signaling. Front Immunol (2017) 8:917. doi: 10.3389/fimmu.2017.00917 28824642 PMC5545922

[56] Boufenzer A Carrasco K Jolly L Brustolin B Di-Pillo E Derive M. Potentiation of NETs Release is Novel Characteristic of TREM-1 Activation and the Pharmacological Inhibition of TREM-1 Could Prevent From the Deleterious Consequences of NETs Release in Sepsis. Cell Mol Immunol (2021) 18(2):452–60. doi: 10.1038/s41423-020-00591-7 PMC8026640 33420354

[57] Jolly L Carrasco K Derive M Lemarié J Boufenzer A Gibot S. Targeted Endothelial Gene Deletion of Triggering Receptor Expressed on Myeloid Cells-1 Protects Mice During Septic Shock. Cardiovasc Res (2018) 114(6):907–18. doi: 10.1093/cvr/cvy018 29361046

[58] Linders J Madhi R Rahman M Mörgelin M Regner S Brenner M. Extracellular Cold-Inducible RNA-Binding Protein Regulates Neutrophil Extracellular Trap Formation and Tissue Damage in Acute Pancreatitis. Lab Invest (2020) 100(12):1618–30. doi: 10.1038/s41374-020-0469-5 32709888

[59] Colonna M. TREMs in the Immune System and Beyond. Nat Rev Immunol (2003) 3(6):445–53. doi: 10.1038/nri1106 12776204

[60] Chung DH Seaman WE Daws MR. Characterization of TREM-3, an Activating Receptor on Mouse Macrophages: Definition of a Family of Single Ig Domain Receptors on Mouse Chromosome 17. Eur J Immunol (2002) 32(1):59–66. doi: 10.1002/1521-4141(200201)32:1<59::AID-IMMU59>3.0.CO;2-U 11754004

[61] Klesney-Tait J Keck K Li X Gilfillan S Otero K Baruah S. Transepithelial Migration of Neutrophils Into the Lung Requires TREM-1. J Clin Invest (2013) 123(1):138–49. doi: 10.1172/JCI64181 PMC3533287 23241959

[62] Tammaro A Scantlebery AML Rampanelli E Borrelli C Claessen N Butter LM. TREM1/3 Deficiency Impairs Tissue Repair After Acute Kidney Injury and Mitochondrial Metabolic Flexibility in Tubular Epithelial Cells. Front Immunol (2019) 10:1469. doi: 10.3389/fimmu.2019.01469 31354698 PMC6629955

[63] Molloy EJ. Triggering Receptor Expressed on Myeloid Cells (TREM) Family and the Application of its Antagonists. Recent Pat Antiinfect Drug Discov (2009) 4(1):51–6. doi: 10.2174/157489109787236292 19149696

[64] Hommes TJ Hoogendijk AJ Dessing MC Van't Veer C Florquin S Colonna M. Triggering Receptor Expressed on Myeloid Cells-1 (TREM-1) Improves Host Defence in Pneumococcal Pneumonia. J Pathol (2014) 233(4):357–67. doi: 10.1002/path.4361 24752755

[65] Hommes TJ Dessing MC Veer C Florquin S Colonna M de Vos AF. Role of Triggering Receptor Expressed on Myeloid Cells-1/3 in Klebsiella-Derived Pneumosepsis. Am J Respir Cell Mol Biol (2015) 53(5):647–55. doi: 10.1165/rcmb.2014-0485OC 25860078

[66] Washington AV Schubert RL Quigley L Disipio T Feltz R Cho EH. A TREM Family Member, TLT-1, Is Found Exclusively in the Alpha-Granules of Megakaryocytes and Platelets. Blood (2004) 104(4):1042–7. doi: 10.1182/blood-2004-01-0315 15100151

[67] Washington AV Gibot S Acevedo I Gattis J Quigley L Feltz R. TREM-Like Transcript-1 Protects Against Inflammation-Associated Hemorrhage by Facilitating Platelet Aggregation in Mice and Humans. J Clin Invest (2009) 119(6):1489–501. doi: 10.1172/JCI36175 PMC2689104 19436112

[68] Derive M Boufenzer A Bouazza Y Groubatch F Alauzet C Barraud D. Effects of a TREM-Like Transcript 1-Derived Peptide During Hypodynamic Septic Shock in Pigs. Shock (2013) 39(2):176–82. doi: 10.1097/SHK.0b013e31827bcdfb 23324887

[69] Li H Guo S Yan L Meng C Hu Y He K. Expression and Purification of a Functional Porcine Soluble Triggering Receptor Expressed on Myeloid Cells 1. Anim Biotechnol (2017) 28(4):237–41. doi: 10.1080/10495398.2016.1267016 28631997

[70] Yang C Zhao J Lin L Pan S Fu L Han L. Targeting TREM-1 Signaling in the Presence of Antibiotics Is Effective Against Streptococcal Toxic-Shock-Like Syndrome (STSLS) Caused by Streptococcus Suis. Front Cell Infect Microbiol (2015) 5:79. doi: 10.3389/fcimb.2015.00079 26618144 PMC4641895

[71] Wang F Liu S Wu S Zhu Q Ou G Liu C. Blocking TREM-1 Signaling Prolongs Survival of Mice With Pseudomonas Aeruginosa Induced Sepsis. Cell Immunol (2012) 272(2):251–8. doi: 10.1016/j.cellimm.2011.10.006 22055202

[72] Horst SA Linnér A Beineke A Lehne S Höltje C Hecht A. Prognostic Value and Therapeutic Potential of TREM-1 in Streptococcus Pyogenes- Induced Sepsis. J Innate Immun (2013) 5(6):581–90. doi: 10.1159/000348283 PMC6741602 23571837

[73] Gibot S Kolopp-Sarda MN Bene MC Bollaert PE Lozniewski A Mory F. A Soluble Form of the Triggering Receptor Expressed on Myeloid Cells-1 Modulates the Inflammatory Response in Murine Sepsis. J Exp Med (2004) 200(11):1419–26. doi: 10.1084/jem.20040708 PMC2211948 15557347

[74] Qian L Weng XW Chen W Sun CH Wu J. TREM-1 as a Potential Therapeutic Target in Neonatal Sepsis. Int J Clin Exp Med (2014) 7(7):1650–8. PMC4132125 25126161

[75] Shi X Zhang Y Wang H Zeng S. Effect of Triggering Receptor Expressed on Myeloid Cells 1 (TREM-1) Blockade in Rats With Cecal Ligation and Puncture (CLP)-Induced Sepsis. Med Sci Monit (2017) 23:5049–55. doi: 10.12659/MSM.904386 PMC5665857 29059148

[76] Gibot S Alauzet C Massin F Sennoune N Faure GC Bene MC. Modulation of the Triggering Receptor Expressed on Myeloid Cells-1 Pathway During Pneumonia in Rats. J Infect Dis (2006) 194(7):975–83. doi: 10.1086/506950 16960786

[77] Sigalov AB. A Novel Ligand-Independent Peptide Inhibitor of TREM-1 Suppresses Tumor Growth in Human Lung Cancer Xenografts and Prolongs Survival of Mice With Lipopolysaccharide-Induced Septic Shock. Int Immunopharmacol (2014) 21(1):208–19. doi: 10.1016/j.intimp.2014.05.001 PMC4088342 24836682

[78] Gibot S Jolly L Lemarié J Carrasco K Derive M Boufenzer A. Triggering Receptor Expressed on Myeloid Cells-1 Inhibitor Targeted to Endothelium Decreases Cell Activation. Front Immunol (2019) 10:2314. doi: 10.3389/fimmu.2019.02314 31632399 PMC6779727

[79] Denning NL Aziz M Diao L Prince JM Wang P. Targeting the eCIRP/TREM-1 Interaction With a Small Molecule Inhibitor Improves Cardiac Dysfunction in Neonatal Sepsis. Mol Med (2020) 26(1):121. doi: 10.1186/s10020-020-00243-6 33276725 PMC7716442

[80] Sharapova TN Romanova EA Chernov AS Minakov AN Kazakov VA Kudriaeva AA. Protein PGLYRP1/Tag7 Peptides Decrease the Proinflammatory Response in Human Blood Cells and Mouse Model of Diffuse Alveolar Damage of Lung Through Blockage of the TREM-1 and TNFR1 Receptors. Int J Mol Sci (2021) 22(20):11213. doi: 10.3390/ijms222011213 34681871 PMC8538247

[81] Derive M Boufenzer A Gibot S. Attenuation of Responses to Endotoxin by the Triggering Receptor Expressed on Myeloid Cells-1 Inhibitor LR12 in Nonhuman Primate. Anesthesiology (2014) 120(4):935–42. doi: 10.1097/ALN.0000000000000078 24270127

[82] François B Wittebole X Ferrer R Mira JP Dugernier T Gibot S. Nangibotide in Patients With Septic Shock: A Phase 2a Randomized Controlled Clinical Trial. Intensive Care Med (2020) 46(7):1425–37. doi: 10.1007/s00134-020-06109-z 32468087

[83] Cuvier V Lorch U Witte S Olivier A Gibot S Delor I. A First-in-Man Safety and Pharmacokinetics Study of Nangibotide, a New Modulator of Innate Immune Response Through TREM-1 Receptor Inhibition. Br J Clin Pharmacol (2018) 84(10):2270–9. doi: 10.1111/bcp.13668 PMC6138490 29885068

[84] Gómez-Piña V Soares-Schanoski A Rodríguez-Rojas A Del Fresno C García F Vallejo-Cremades MT. Metalloproteinases Shed TREM-1 Ectodomain From Lipopolysaccharide-Stimulated Human Monocytes. J Immunol (2007) 179(6):4065–73. doi: 10.4049/jimmunol.179.6.4065 17785845

[85] Palazzo SJ Simpson T Schnapp LM. Triggering Receptor Expressed on Myeloid Cells Type 1 as a Potential Therapeutic Target in Sepsis. Dimens Crit Care Nurs (2012) 31(1):1–6. doi: 10.1097/DCC.0b013e31823a5298 22156803 PMC4754668

[86] Jedynak M Siemiatkowski A Mroczko B Groblewska M Milewski R Szmitkowski M. Soluble TREM-1 Serum Level can Early Predict Mortality of Patients With Sepsis, Severe Sepsis and Septic Shock. Arch Immunol Ther Exp (Warsz) (2018) 66(4):299–306. doi: 10.1007/s00005-017-0499-x 29282483 PMC6061141

[87] Chang W Peng F Meng SS Xu JY Yang Y. Diagnostic Value of Serum Soluble Triggering Expressed Receptor on Myeloid Cells 1 (sTREM-1) in Suspected Sepsis: A Meta-Analysis. BMC Immunol (2020) 21(1):2. doi: 10.1186/s12865-020-0332-x 31931717 PMC6958609

[88] Gibot S Massin F. Soluble Form of the Triggering Receptor Expressed on Myeloid Cells 1: An Anti-Inflammatory Mediator? Intensive Care Med (2006) 32(2):185–7. doi: 10.1007/s00134-005-0018-0 16450101

[89] Jolly L Carrasco K Salcedo-Magguilli M Garaud JJ Lambden S van der Poll T. sTREM-1 Is a Specific Biomarker of TREM-1 Pathway Activation. Cell Mol Immunol (2021) 18(8):2054–6. doi: 10.1038/s41423-021-00733-5 PMC8322270 34282296

[90] Yang C Chen B Zhao J Lin L Han L Pan S. TREM-1 Signaling Promotes Host Defense During the Early Stage of Infection With Highly Pathogenic Streptococcus Suis. Infect Immun (2015) 83(8):3293–301. doi: 10.1128/IAI.00440-15 PMC4496610 26056380

[91] Tan C Gurien SD Royster W Aziz M Wang P. Extracellular CIRP Induces Inflammation in Alveolar Type II Cells *via* TREM-1. Front Cell Dev Biol (2020) 8:579157. doi: 10.3389/fcell.2020.579157 32984356 PMC7484489

[92] Sigalov AB. SCHOOL of Nature: Ligand-Independent Immunomodulatory Peptides. Drug Discov Today (2020) 25(8):1298–306. doi: 10.1016/j.drudis.2020.05.005 PMC7217646 32405248

[93] Sigalov AB. Commentary: Triggering Receptor Expressed on Myeloid Cells-1 Inhibitor Targeted to Endothelium Decreases Cell Activation. Front Immunol (2020) 11:173. doi: 10.3389/fimmu.2020.00173 32117302 PMC7026307

[94] Gattis JL Washington AV Chisholm MM Quigley L Szyk A McVicar DW. The Structure of the Extracellular Domain of Triggering Receptor Expressed on Myeloid Cells Like Transcript-1 and Evidence for a Naturally Occurring Soluble Fragment. J Biol Chem (2006) 281(19):13396–403. doi: 10.1074/jbc.M600489200 16505478

[95] Parent M Clarot I Gibot S Derive M Maincent P Leroy P. One-Week *In Vivo* Sustained Release of a Peptide Formulated Into *In Situ* Forming Implants. Int J Pharm (2017) 521(1-2):357–60. doi: 10.1016/j.ijpharm.2017.02.046 28232200

[96] Advertisement Feature in Nature, Under Biopharma Dealmakers. Retrieved at: https://www.nature.com/articles/d43747-020-00839-1.

[97] National Library of Medicine (U.S.) Efficacy, Safety and Tolerability of Nangibotide in Patients With Septic Shock (ASTONISH) United States: NIH U.S. National Library of Medicine (2019).

[98] Leong K Gaglani B Khanna AK McCurdy MT. Novel Diagnostics and Therapeutics in Sepsis. Biomedicines (2021) 9(3):311. doi: 10.3390/biomedicines9030311 33803628 PMC8003067

[99] Brynjolfsson SF Magnusson MK Kong PL Jensen T Kuijper JL Håkansson K. An Antibody Against Triggering Receptor Expressed on Myeloid Cells 1 (TREM-1) Dampens Proinflammatory Cytokine Secretion by Lamina Propria Cells From Patients With IBD. Inflamm Bowel Dis (2016) 22(8):1803–11. doi: 10.1097/MIB.0000000000000822 27243593

[100] Ulloa L Brunner M Ramos L Deitch EA. Scientific and Clinical Challenges in Sepsis. Curr Pharm Des (2009) 15(16):1918–35. doi: 10.2174/138161209788453248 PMC3098530 19519432

[101] Ranieri VM Thompson BT Barie PS Dhainaut JF Douglas IS Finfer S. Drotrecogin Alfa (Activated) in Adults With Septic Shock. N Engl J Med (2012) 366(22):2055–64. doi: 10.1056/NEJMoa1202290 22616830

[102] Cavaillon JM Singer M Skirecki T. Sepsis Therapies: Learning From 30 Years of Failure of Translational Research to Propose New Leads. EMBO Mol Med (2020) 12(4):e10128. doi: 10.15252/emmm.201810128 32176432 PMC7136965

